# Cytokine Networks in Lichen Sclerosus: A Roadmap for Diagnosis and Treatment?

**DOI:** 10.3390/ijms26094315

**Published:** 2025-05-01

**Authors:** Alessia Paganelli, Dario Didona, Emanuele Scala

**Affiliations:** 1 Dermatology Unit, IDI-IRCCS Istituto Dermopatico dell’Immacolata, 00167 Rome, Italy; a.paganelli@idi.it; 2 Rare Diseases Unit, IDI-IRCCS Istituto Dermopatico dell’Immacolata, 00167 Rome, Italy; 3 Laboratory of Experimental Immunology, IDI-IRCCS Istituto Dermopatico dell’Immacolata, 00167 Rome, Italy

**Keywords:** lichen sclerosus, pathogenesis, cytokines, TGFβ, ECM1, chemokines

## Abstract

Lichen sclerosus (LS) is a chronic inflammatory skin disorder primarily affecting the anogenital region, leading to symptoms such as itching, pain, and sexual dysfunction, all of which significantly impact patients’ quality of life. Due to the non-specific nature of its early symptoms, diagnosis is often delayed. This review examines the cytokine networks involved in LS, with a focus on immune activation, the role of T-helper (Th)1 cells, and the interaction between inflammatory mediators and the extracellular matrix, particularly in fibrosis. By providing an updated understanding of LS immunopathogenesis, this review highlights key mediators involved in disease progression and offers insights into personalized treatment strategies that may improve patient outcomes. Additionally, current therapeutic approaches and future directions in LS management are discussed.

## 1. Introduction

Lichen sclerosus (LS) is a chronic inflammatory skin disease that typically involves the anogenital area. Local itching and pain lead to sexual and urinary dysfunction, deeply impacting patients’ quality of life [1,2]. Usually, LS begins with hypochromic changes in the genital mucosa, but it can eventually lead to significant architectural alteration of the genital area [3]. Due to the non-specific nature of the most bothersome symptoms and of the initial clinical manifestations, diagnosis is often delayed [3,4].

LS can occur at every age and in both sexes. The male-to-female ratio varies between 1:3 and 1:10 [2]. Not surprisingly, significant variations in the incidence and prevalence of the disease have been described according to sex and age. LS prevalence varies from approximately 0.1% in the pediatric population up to 3% in women older than 80 [2,5]. A recent study reported LS to occur in 1.6% of women by the age of 80 [6], while in men the proportion was estimated to be significantly lower (0.07%) [7]. Despite LS being mostly diagnosed in postmenopausal women, the disease is demonstrated to begin before the menopause in at least half of cases [4]. However, overall LS prevalence is probably underestimated because LS is underdiagnosed [4].

The diagnosis is mostly clinical, but a skin biopsy is often performed to confirm the diagnosis or to rule out cutaneous malignancies possibly arising in the setting of LS. Indeed, a vulval or penile biopsy is not usually performed in the pediatric population because there is no risk of dysplasia or cancer in prepubertal children justifying such an invasive procedure [8]. Furthermore, histopathological analysis of the foreskin after circumcision can reveal non-recognized LS in about 38% of cases [9]. The delay in diagnosis and lack of targeted therapies make the treatment of LS particularly tricky, even for the most skilled clinicians. As such, wider comprehension of the key pathogenic mechanisms is crucial for the development of new therapeutic strategies in this setting.

The aim of the present review is to provide new insights into LS pathogenesis, shedding light on possible mechanisms underlying the immune activation and fibrosis that strongly characterize the disease. In particular, we focus on the cytokine milieu and on the interaction between inflammatory soluble mediators and extracellular matrix (ECM) components.

## 2. Materials and Methods

This is a narrative review. Thus, no predefined inclusion and exclusion criteria were set prior to starting the evaluation of electronic sources. The PubMed and SCOPUS electronic databases were used for our search. The key terms used according to our search strategy were “lichen sclerosus” and “cytokines” or “chemokines” or “interleukin” or “ECM1”. The database search was performed from inception to present. The article selection was conducted independently by A.P., D.D., and E.S., with the final list being agreed between the three independent researchers before manuscript preparation. The following PICO (Population, Intervention or Exposure, Comparison, Outcome) algorithm was applied in the present review: (i) Population: patients affected by lichen sclerosus; (ii) Intervention: assessment of cytokine dysregulation as a possible pathogenetic mechanism; (iii) Comparator: other possible pathways implicated in disease development; (iv) Outcome: identification of the most important cytokine pathways dysregulated in the setting of LS and identification of potential new therapeutic targets.

## 3. Diagnosis

**Figure 1:**
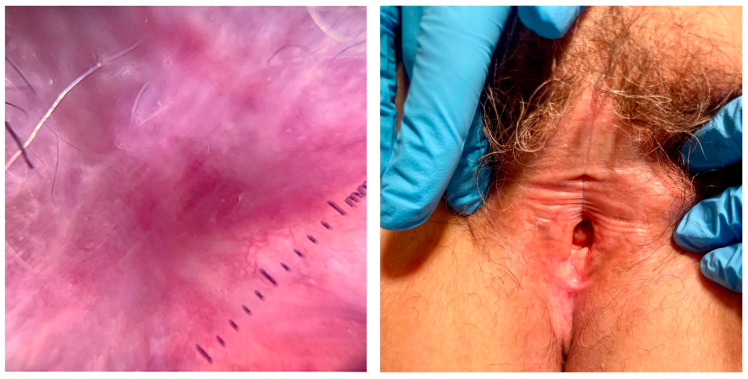
Dermoscopic and clinical pictures of vulvar LS. Dermoscopy (**left panel**) shows white structureless areas, chrysalis-like structures, and linear and/or reticular vessels. Clinically (**right pane**l), advanced LS presents with fusion of the labia minora and labia majora, clitoral hooding, and a reduction of the vaginal introitus.

As already anticipated, the diagnosis often relies on the presence of typical clinical findings (Figure 1). The clinical features of LS are quite variable. While white plaques represent the hallmarks of the disease, painful erosions and rhagades can also occur [4]. Other signs commonly associated with LS include erythema, ecchymoses, and dyschromic changes [10]. LS progression leads to sclerosis, scarring, and the formation of synechiae [4]. In women, these features are usually associated with pruritus and dyspareunia, while men often present with phimosis, leading to erectile dysfunction and eventually pain [4]. Discomfort and a burning sensation are also not uncommon in LS patients [11]. Lastly, tearing and vaginal discharge are sometimes reported in female patients [12].

As for disease localization, a recent systematic review focused on confirmed LS reported that it mostly affects the genital area (77% of females, 99% of males) [12]. The authors also underscored increased rates of extragenital involvement in female patients. In more detail, the extremities and the anal area were found to be involved in 7% and 8% of cases, respectively, in female patients, while involvement of these areas was described in <1% of men with LS [12]. Similarly, the involvement of the trunk was reported in up to 2% of females and in <1% of males with LS [12].

Relatively frequent complications include sexual, urinary, and psychological impairment [12,13,14]. Sexual dysfunction was found to be the most frequent LS-related complication in a cohort of female patients (56%), while the most frequently reported complications in male patients are urinary obstruction or retention (49%) [12]. However, while sexual dysfunction is also rather common in male patients (28%), urinary obstruction/retention was reported in less than 1% of women with LS [12].

The literature suggests that LS may increase the risk of developing squamous cell carcinoma (SCC), with chronic inflammation possibly playing a key triggering role [15]. In LS patients, the risk of developing genital SCC seems to be slightly increased compared to the general population and has been estimated to be approximately 4–5% [2]. Interestingly, in a prospective cohort study, patients compliant with a topical corticosteroid (TCS) maintenance regimen did not develop vulvar SCC, while those with only partial compliance had an overall incidence of 4.7% of vulvar intraepithelial neoplasia (VIN) and SCC. Taken together, these findings suggest that therapy-driven modulation of the LS inflammatory background may modify the course of the disease, playing a role in avoiding the development of vulvar SCC [16].

LS diagnosis could be difficult in early stages with exclusive genital involvement, especially in the female population, because LS shares several signs and symptoms with other disorders of the genital area, including candidiasis and/or bacterial infection [8]. Disease progression leads to easier clinical diagnosis in advanced stages; however, currently available treatments are not effective with respect to final-stage fibrotic changes, and early diagnosis is therefore of crucial importance. Indeed, a recent clinico-pathological retrospective study underscored an accuracy of 81.8% of dermatological evaluation in diagnosing LS [9]. As for extragenital disease, LS poses a great diagnostic challenge even for skilled clinicians, possibly resembling conditions like discoid lupus, morphea, vitiligo, hypopigmented mycosis fungoides, graft-vs.-host disease, and even blistering disorders [17]. Due to all these possible differentials, histopathological confirmation is often needed for extragenital forms. Histology also plays a role in helping the diagnosis in milder/initial cases. The most recent guidelines support the notion that a confirmatory biopsy is not always mandatory but is indicated in doubtful clinical cases, refractory lesions, or if a malignant or pre-malignant lesion is suspected [18]. LS histopathological features include epidermal atrophy, compact ortho-/hyperkeratosis, an interface dermatitis with a band-like infiltrate, basal degeneration, dermal hyalinization, and follicular plugging [8,9].

Of note, non-invasive imaging techniques, such as dermoscopy and confocal microscopy, also play an increasingly valuable role in the diagnosis of LS [19]. Dermoscopy can reveal characteristic vascular and pigmentary changes, such as white structureless areas, linear and dotted vessels, and comedo-like openings [20,21]. Reflectance confocal microscopy (RCM) provides high-resolution, in vivo visualization of epidermal and dermal structures, aiding in the identification of features like epidermal atrophy, inflammatory cell infiltration, and disruption of collagen architecture, which are hallmarks of LS [22,23]. These techniques enhance diagnostic accuracy and may reduce the need for invasive biopsies.

## 4. Etiopathogenesis

### 4.1. Pathogenic Events

**Figure 2:**
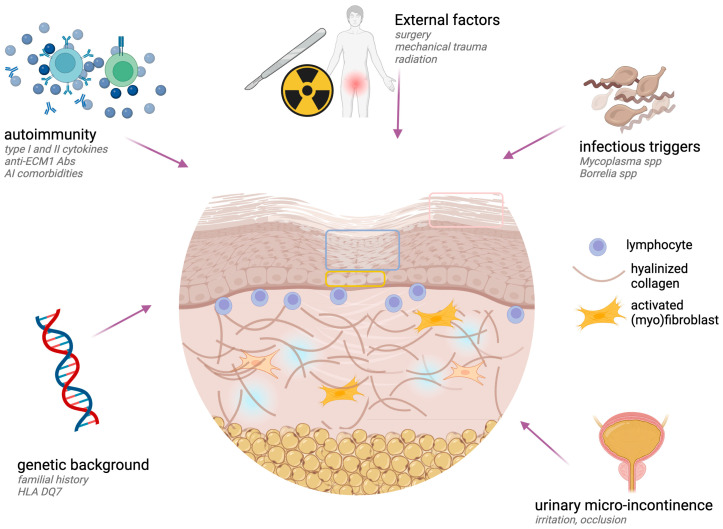
Schematic representation of the main pathogenetic mechanisms in LS. The complex interplay between genetic, immunological, mechanical, physical, and infectious agents leads to the development of LS-specific changes in affected skin: interface dermatitis with inflammatory cells at the dermal–epidermal junction level, derma sclerosis (especially in the upper dermis), epidermal atrophy (blue square), hyperkeratosis (pink square), basal degeneration (yellow square). Created with BioRender.com.

The development of LS is influenced by a complex interaction of genetic and environmental factors, which trigger an abnormal immune response characterized by an imbalance of pro-inflammatory cytokines and dysfunction of regulatory T cells, ultimately leading to alterations in collagen metabolism [24,25]. This complex series of events result in fibrotic changes and scarring of the affected tissues, defining the hallmark features of the disease [26]. An overview of the main pathogenetic mechanisms is provided in Figure 2. From a genetic point of view, a positive family history of LS is reported in between 5.4% and 12% of patients [27,28,29]. In addition, a significant association with specific HLA class-II antigens (HLA DQ7 in particular) has been pointed out in LS patients compared with controls [25,30,31].

The scientific literature on external triggering factors in LS is quite controversial. However, several factors have been reported as possibly playing a role in the development of LS. Pelvic surgery, radiation, repeated mechanical reduction of the foreskin, and genital injury have been thought to induce LS in genetically predisposed individuals [32,33,34].

The role of genital bacterial infections in the pathogenesis of LS is debated. While a population-based case–control study did not find a role for genital bacterial infections in LS development [33], vulvitis and/or urethritis were found to be significantly associated with the occurrence of LS in an epidemiological study on 100 cases of vulvar LS [35]. Both PCR and ELISA detected a significant presence of *Mycoplasma* spp. in LS when compared to controls [18]. Furthermore, *Borrelia* species were identified in 63% of LS cases, with even higher rates in early inflammatory LS cases [36]. Interestingly, according to the current literature, HPV is not related to the development of LS [37,38], therefore reinforcing the notion that inflammation is the primary driver in LS-related SCCs.

Despite the role of urinary occlusion and/or incontinence in the development of LS being supported by several studies, the interplay between this peculiar trigger and the genetic background needs to be further investigated, especially in women [39,40,41]. A role for micro-incontinence, however, has been confirmed in the setting of LS, both clinically and histologically [42,43]. A role for stagnation of urine in contact with the skin and micro-incontinence is further supported by relative sparing of the uncovered areas of the genitals in LS patients [44].

### 4.2. Immune Factors and Cytokine Networks

Immune system dysregulation plays a central role in the pathogenesis of LS, with both humoral and T cell-mediated mechanisms implicated [45]. A significant proportion of LS patients exhibit one or more autoimmune-related conditions, a family history of autoimmune diseases, or the presence of autoantibodies [26]. Common comorbidities include thyroiditis, alopecia areata, vitiligo, and pernicious anemia, while rarer associations include bowel disease, localized scleroderma/morphea, rheumatoid arthritis, systemic lupus erythematosus, and multiple sclerosis.

Pathogenically, LS is characterized by a predominant Th1 immune response, leading to the overproduction of pro-inflammatory cytokines and mediators such as IL-1, IL-7, IL-15, IFN-γ, TNF-α, CD25 (IL-2 receptor), caspase-1, ICAM-1, and its ligand CD11a [17,26,46,47,48,49]. Genetic studies have identified specific IL-1 receptor antagonist polymorphisms linked to disease progression [50]. Czajkowski et al. demonstrated that IL-1α, IL-6, and IFN-γ were detectable, respectively, in early, moderate, and severe stages of penile LS (PLS), with disease progression being closely related to micro-incontinence [48].

The type-1 cytokine cascade is further amplified by the upregulation of chemokine receptors, including CXCR3, CXCL9-11, CCR5, CCL4, and CCL5, which facilitate the recruitment of inflammatory cells to the lesions [46,51].

Despite significant progress in understanding the immunological mechanisms of LS, a definitive disease marker remains elusive, and none of the identified mediators fully explain the underlying cause of pruritus. However, the positive response of extragenital LS to dupilumab suggested that IL-4/IL-13 signaling may also play a role in LS, particularly in cases characterized by pruritus and lichenification [52]. In line with these observations, Carli et al. (1997) [53] first reported increased IL-4 expression in vulvar LS, especially in early lesions. In contrast, IFN-γ staining in LS was minimal (less than 10% infiltrating cells). These findings were found to be extremely specific for LS, since inverse IL-4 and IFN-γ expression patterns were described in vulvar lichen planus (LP). Interestingly, the same study indicated similar staining for transforming growth factor-beta (TGF-β), a key pro-fibrotic mediator, in both LS and LP [53].

A recent Polish study [54] reported significantly elevated levels of IL-17 in the epidermis and upper dermal infiltrates of 20 patients with histologically confirmed VLS compared to age- and sex-matched controls (n = 10). In contrast, a separate study [51] found no significant gene regulation of key factors involved in Th17 (IL-17A, IL-17F, and IL-22R) or Th2 (CCR3 and IL-4) responses in LS. These conflicting findings underscore the need for further research to clarify the role of IL-17 in LS pathogenesis and to determine whether it represents a potential therapeutic target.

Not surprisingly, the exaggerated inflammatory response in LS is also attributed to a reduction in regulatory T cell (Treg) activity. This is further accompanied by lower levels of IL-10, without a parallel decrease in TGF-β levels [45,47]. IL-10, a critical anti-inflammatory mediator, plays a vital role in modulating immune responses, whereas TGF-β is essential for Treg differentiation and function [55,56]. The diminished Treg activity in LS has been linked to overexpression of microRNA-155 (miR-155) and reduced Foxp3+ Treg levels in LS lesions [51,57]. In a mouse model, artificially increasing miR-155 expression led to an altered Treg cell phenotype, impairing Treg suppressive function [58]. Similarly, another study showed that elevated miR-155 levels in CD4+ T cells compromised Treg-mediated suppression [59]. Collectively, these immune alterations not only sustain inflammation but also foster a persistent autoimmune environment, driving autoantibody production, which exacerbates tissue damage and perpetuates the cycle of disease progression (see Section 4.3).

Recently, an emerging role in LS pathogenesis has been demonstrated for factors secreted by keratinocytes and fibroblasts, such as Dkk-1, GDF-15, IGFBP-2, and CHI3L1 [60]. These findings suggest that different resident skin cell types actively contribute to LS pathogenesis. However, more studies are required to better understand the roles and interactions of keratinocytes and fibroblasts in the development of VLS.

An overview of the key cytokines implicated in LS and their effects on immune function is presented in Table 1.

### 4.3. TGF-β and ECM1: A Complex Interplay in Fibrosis and Tissue Remodeling

ECM1 was first identified in 1994 in mouse osteogenic stromal cell lines [73]. Since then, ECM1 has been detected in various human tissues, including the skin, liver, heart, skeletal muscle, and placenta [74]. The discovery in 2002 that mutations in the ECM1 gene cause lipoid proteinosis significantly advanced our understanding of ECM1’s role in human skin physiology and pathology [75]. Lipoid proteinosis is a rare autosomal recessive genodermatosis characterized by widespread hyalinization of the dermis in both skin and mucosae, often leading to scarring [76].

Lipoid proteinosis exhibits several histopathological features that overlap with LS, including dermal hyalinosis and fibrosis [77]. Clinically, it resembles a particularly severe form of LS, with widespread extragenital involvement. Notably, the similarities between lipoid proteinosis and LS prompted a groundbreaking study by Oyama and collaborators in 2003 [77]. Their research demonstrated the presence of anti-ECM1 autoantibodies in 75% of patients affected by vulvar LS. Subsequent studies confirmed these findings in male genital LS [78]. Interestingly, these autoantibodies appeared to be LS-specific, as they were absent in healthy subjects and other sclerosing and/or autoimmune skin diseases, such as lupus, systemic sclerosis, and bullous pemphigoid. However, these studies did not determine whether the antibodies were pathogenetic or merely an epiphenomenon. Nonetheless, these discoveries spurred further research into the pathogenetic mechanisms of LS related to ECM1 alterations.

**Figure 3:**
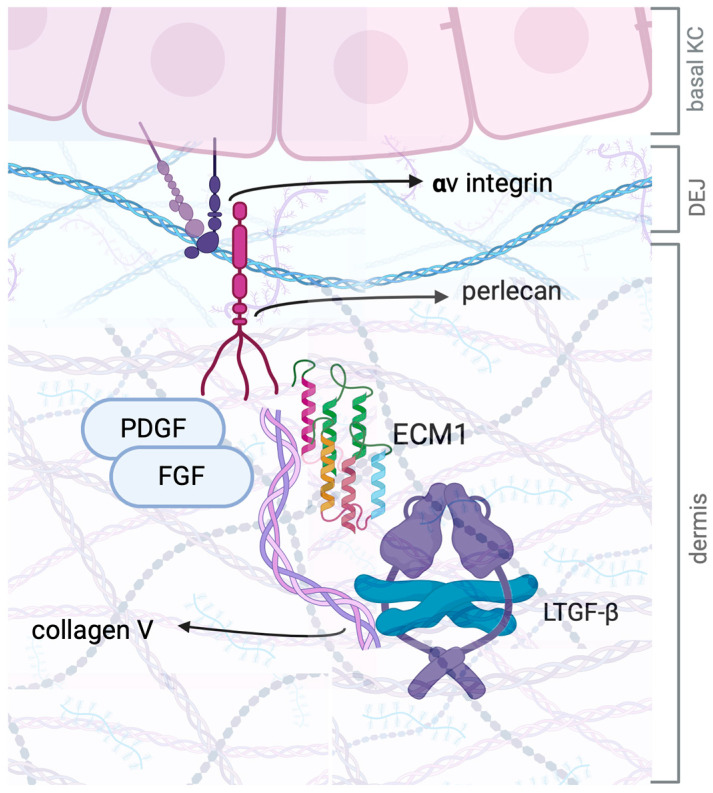
Schematic representation of the main interactions of ECM1 in the upper dermal compartment. In the dermis, ECM1 is mostly present in the upper dermis and at the DEJ (dermal–epidermal junction) level. ECM1 functions as a glue for dermal proteins, including collagen and proteoglycans. ECM1 is also crucial for maintaining DEJ integrity through binding with perlecan and integrins on basal keratinocytes. Lastly, ECM1 also colocalizes with collagen V and shares with this molecule a key role in modulating the activation and the release of pro-fibrotic growth factors hidden in the dermis. KC: keratinocyte; PDGF: platelet-derived growth factor; FGF: fibroblast growth factor; ECM1: extracellular matrix protein 1; LTGF: latent transforming growth factor β. Created with BioRender.com.

In the skin, both keratinocytes and fibroblasts contribute to ECM1 production, with the protein being predominantly expressed in the epidermis and upper dermis [74]. More specifically, ECM1 is mainly located in basal keratinocytes, where it is known to play a role in keratinocyte differentiation [74]. Within the dermis, ECM1 binds to proteoglycans such as perlecan and possibly functions as a “glue” for dermal proteins [79] (see Figure 3). As a result, any disruption in these associations may lead to architectural changes in the upper dermis and at the dermal–epidermal junction (DEJ). Furthermore, perlecan colocalizes with ECM1 at the basal membrane and interacts with fibronectin, laminin, collagen, elastic fibers, platelet-derived growth factor (PDGF), and fibroblast growth factor (FGF) [74]. PDGF and FGF also bind to phospholipid scramblase 1 (PLSCR-1), a calcium-dependent phospholipid transporter that in turn interacts directly with ECM1 [80].

Both FGF and PDGF contribute significantly to fibrosis by promoting fibroblast proliferation, migration, and extracellular matrix deposition, ultimately leading to tissue scarring and dysfunction [81]. Any disruption in these interactions could contribute to the hyaline abnormalities characteristic of LS. Despite ECM1 being predominantly produced in the basal layers of the epidermis, researchers have recently investigated ECM1-related alterations in dermal fibroblasts through gene silencing [82]. Their study identified 3035 differentially expressed genes, with most upregulated genes encoding proteins involved in fibrosis and ECM organization, including transforming growth factor-beta receptor (TGFBR), PDGF, collagen, fibronectin, and laminins. The same study also revealed direct interactions between ECM1 and collagen VII at the DEJ, which may explain the abnormal collagen-7 expression observed in LS. These findings led to the conclusion that ECM1-knockout fibroblasts exhibited phenotypic similarities to fibroblast alterations found in LS.

Reduced ECM1 expression in vulvar LS has also been associated with decreased elastic fiber deposition, while an inverse correlation has been observed between collagen V and ECM1 content in LS-affected skin [83]. The dense clustering of collagen V within the hyalinized zone of the upper dermis in LS suggests that collagen V may trigger ECM1 dysregulation, playing a key role in disease pathogenesis [83]. As for the possible mechanisms, the a1(V)-N-pro-peptide binds crucial regulators of ECM remodeling, such as TGF-b1, matrix metalloproteinase 2 (MMP-2), and tissue inhibitor of metalloproteinase-1 (TIMP-1) [84]. Thus, COLV could be involved in controlling TGF-β and metalloproteinase release in the fibrotic process [85]. However, TGF-β induces upregulation of *COL5a1* and *COL5a2* genes, therefore inducing increased collagen V deposition in the dermis [85].

Beyond its structural and binding properties, ECM1 also serves as a critical regulator of tissue remodeling by maintaining the latency of deposited latent TGF-β [86]. The latter is secreted in a latent form, associated with its N-terminal pro-peptide, also known as the latency-associated peptide (LAP), which renders the growth factor inactive. Various molecules, including proteases, reactive oxygen species, and αV-integrins, can induce latent TGF-β activation [87].

TGF-β plays a central role in fibrosis by stimulating fibroblast activation, myofibroblast differentiation, and excessive ECM deposition, contributing to tissue scarring and dysfunction [88]. Multiple studies have demonstrated increased TGF-β levels in LS. Specifically, elevated levels of TGF-β, its receptors (TGFBRs), and matrix metalloproteinases (MMPs) have been detected in LS tissues through both RT-PCR and immunohistochemistry [89]. Studies on foreskin samples from boys with LS-related phimosis confirmed increased TGF-β expression, as did immunohistochemical analyses of vulvar LS tissues [53]. However, the exact role of TGF-β in LS pathogenesis remains controversial. In fact, recent single-cell sequencing and spatial transcriptomic studies have suggested an unexpected reduction in TGF-β signaling pathway activation in LS-lesional skin [90].

Given TGF-β’s central role in fibrosis, the disruption of the balance between its latent and active forms is likely involved in fibrosing disorders such as LS. Recent hepatological studies have provided insights into ECM1’s role in maintaining latent TGF-β homeostasis. Mechanistically, ECM1 inhibits αV-integrin-mediated latent TGF-β activation by interfering with its arginine–glycine–aspartic acid (RGD) motif [91]. This mechanism has already been explored in liver fibrosis, where chronic liver disease is characterized by decreased ECM1 release from hepatocytes, leading to increased latent TGF-β activation. Similar processes may contribute to fibrosis in LS, suggesting that ECM1 alterations not only influence ECM organization but also impact TGF-β activation, thereby exacerbating fibrotic changes.

In summary, ECM1 plays a multifaceted role in maintaining skin integrity, interacting with a range of ECM components and growth factors. Its involvement in LS pathogenesis is supported by both genetic and immunological evidence, with studies highlighting ECM1 downregulation, the presence of anti-ECM1 autoantibodies, and its impact on fibrosis-related pathways. The interplay between ECM1, perlecan, PDGF, FGF, collagen, and TGF-β provides a complex yet crucial framework for understanding how ECM1 dysregulation contributes to fibrotic disorders. Further research into ECM1’s regulatory mechanisms may pave the way for novel therapeutic strategies aimed at modulating fibrosis and restoring tissue homeostasis in LS and other fibrosing conditions.

## 5. Therapeutic Management: Current Guidelines and State of the Art

The management of LS is centered on topical corticosteroids (TCSs) as first-line therapy, particularly high-potency corticosteroids like clobetasol propionate [92]. These have been shown to effectively reduce inflammation, improve symptoms, and prevent disease progression [17,93]. Long-term maintenance therapy is often required to prevent relapses [2,94]. In patients with corticosteroid resistance or intolerance, topical calcineurin inhibitors (TCIs), such as tacrolimus and pimecrolimus, serve as an alternative. TCIs work by inhibiting T cell activation and reducing inflammatory cytokine production, offering a corticosteroid-sparing option for long-term management [93,95].

Emollients and hydrating agents as well as vitamin E-containing ointments are considered safe and valid adjunctive tools, used either in combination or alone as maintenance treatments [93].

A recent study that aimed to compare the long-term effectiveness of vitamin E and emollients found no significant differences between the two therapeutic options; the authors therefore concluded that both may be considered in maintaining LS remission [96].

As for other available treatments, the most recent guidelines recommend against topical hormone preparations due to the lack of significant clinical benefits from the use of topical progesterone and/or progesterone as a maintenance therapy [93,97].

For more severe or refractory LS cases, systemic immunosuppressive therapies, including cyclosporine, mycophenolate methotrexate, and biologics, have been considered, though evidence supporting their efficacy is still limited [2,93,98]. The most recent guidelines, in fact, only consider methotrexate and acitretin as recommended systemic treatments in adult patients with refractory genital LS [93]. Early observations and initial case reports have explored the use of biologic therapies for treatment, with promising results for dupilumab being reported in LS-related itching [52,93].

Phototherapy, particularly using ultraviolet (UV) A light, is a recognized and effective treatment for LS, with several studies, including prospective controlled trials, supporting its use in sclerosing skin conditions [93,99]. Another alternative non-pharmacological approach is photodynamic therapy (PDT), which has been investigated for vulvar LS. PDT involves applying a photosensitizing agent followed by light exposure, inducing immune modulation and targeted destruction of affected tissue [100]. Systematic reviews suggest that PDT improves both clinical and histopathological LS features, though protocols require optimization [100,101].

Recent advancements in regenerative medicine have highlighted the potential of platelet-rich plasma (PRP) and adipose-derived stem cell (ADSC) therapy. PRP contains growth factors such as platelet-derived growth factor (PDGF) and fibroblast growth factor (FGF), which promote tissue regeneration and remodeling [102]. ADSCs, which possess immunomodulatory and regenerative properties, have shown promise in reducing fibrosis, improving skin elasticity, and restoring extracellular matrix (ECM) integrity in LS-affected tissues. Though preliminary studies indicate positive outcomes, larger controlled trials are necessary to establish their efficacy [103,104].

Surgical interventions are generally reserved for severe LS cases with extensive scarring or functional impairment [3]. CO_2_ laser therapy and surgical excision may be employed to release adhesions or restore anatomical function, though recurrence is common, and postoperative care is essential to prevent disease progression [39,93,105]. Given LS’s chronic and relapsing nature, an interdisciplinary approach—including dermatologists, gynecologists, and urologists—is crucial for optimal management [93,105].

## 6. Recent Advances and Future Perspectives

Emerging evidence suggests that LS pathogenesis involves dysregulation of the ECM1-TGFβ axis, contributing to increased fibrosis and dermal remodeling [77]. ECM1 normally plays a role in maintaining basement membrane structure and ECM homeostasis [74]. Its deficiency has been associated with uncontrolled TGFβ activation, which in turn promotes fibroblast activation, excessive collagen deposition, and scarring [91]. Given the central role of TGFβ in fibrotic remodeling, targeting this pathway presents potential therapeutic opportunities. TGFβ inhibitors, including monoclonal antibodies and small-molecule inhibitors, have been explored in fibrotic diseases, though systemic inhibition poses risks such as impaired wound healing and immune dysfunction [98,106,107]. Biologic therapies directed against specific cytokines involved in fibrosis and inflammation are under preliminary investigation and may represent an additional future strategy for refractory or severe cases of LS. An alternative approach may involve enhancing ECM1 expression or function, thereby preventing excessive TGFβ activation. Future therapeutic directions may focus on targeting αV-integrins and/or collagen V, with subsequent modulation of the ECM1-TGFβ axis, paving the way for innovative, targeted treatments aimed at preserving ECM integrity and reducing fibrosis in LS.

Regenerative therapies, including PRP and ADSCs, may exert beneficial effects by modulating TGFβ signaling. PRP contains growth factors (PDGF and FGF) that could counteract fibrosis by promoting ECM remodeling. Mesenchymal stem cells in particular have been demonstrated to modulate ECM1 upregulation, which may be relevant for LS treatment in terms of anti-fibrotic properties [108].

Another promising avenue is the drug repurposing of Janus kinase (JAK) inhibitors, which have demonstrated efficacy in various inflammatory dermatoses and are now available in topical formulations (e.g., ruxolitinib) for vitiligo and atopic dermatitis. Their application in LS could offer a targeted, localized immunomodulatory effect, potentially minimizing systemic adverse events, as recent studies show promising results in the setting of LS [98,109,110]. However, further clinical trials are needed to validate modulation of the JAK-STAT signaling pathway as an effective therapeutic tool in the setting of LS.

Nonetheless, innovation in the context of LS extends beyond therapeutic strategies. In fact, early diagnosis and accurate monitoring of disease progression are essential for maximizing treatment efficacy. However, several challenges remain, including (i) the lack of early biomarkers indicative of therapeutic response or failure, (ii) ethical and economic limitations preventing routine or widespread use of invasive techniques such as biopsies, (iii) the need to identify minimal inflammatory and fibrotic changes before clinically evidenced architectural changes in the external genitalia occur, and (iv) the limited ability of clinical examination alone to visualize structures beyond the epidermal surface.

Non-invasive imaging technologies such as dermoscopy and reflectance confocal microscopy (RCM) have opened new avenues for visualizing subclinical features and characterizing skin changes in vivo with high resolution. Furthermore, advances in non-invasive imaging technologies, particularly line-field confocal optical coherence tomography (LC-OCT), hold considerable potential for improving early diagnosis, objectively monitoring disease progression, and assessing therapeutic response in LS, thereby facilitating more precise and individualized patient care.

The use of software tools and open-access databases to support biomarker validation in lichen sclerosus remains in its early stages, while there is growing interest in leveraging bioinformatics to identify and validate molecular targets for improved diagnosis and personalized therapy; in the future, integrating multi-omics data and artificial intelligence-driven analyses could significantly accelerate biomarker discovery and enable more precise patient assessment.

Last but not least, emerging applications of artificial intelligence are poised to significantly enhance the early diagnosis of lichen sclerosus, enable more accurate patient stratification based on disease severity and progression risk, and support the development of personalized therapeutic approaches tailored to individual patient profiles.

## 7. Discussion

LS is a chronic inflammatory skin disorder that significantly affects patient health and quality of life, particularly impairing sexual and urinary function [14,42]. Due to its potential for irreversible damage to genital and perianal areas, early diagnosis and intervention are essential in preventing long-term complications. While typically diagnosed in postmenopausal women, LS can also manifest earlier, including in childhood or adolescence [6,17].

Hallmark signs such as hypopigmentation, sclerosis, and scarring are common in advanced LS [10], but early-stage presentations can be subtle and are often misdiagnosed. As a result, delays in treatment are not an uncommon finding in LS. Moreover, although traditionally considered rarer in men, an increasing number of diagnoses in circumcision specimens suggests that LS may be more prevalent in males than previously recognized [111].

Emerging evidence highlights the importance of non-invasive diagnostic methods such as dermoscopy and RCM in doubtful cases. These techniques enhance diagnostic accuracy and enable early detection without the need for invasive biopsies. Early detection is crucial for preventing disease progression, improving patient outcomes, and minimizing the risk of associated complications. Given the challenges of managing refractory cases and delayed diagnoses, there is a pressing need for novel, targeted therapies that address the underlying inflammatory pathways and ECM remodeling processes driving LS pathology.

A key characteristic of LS is immune dysregulation, especially an exaggerated Th1 response, which results in elevated levels of pro-inflammatory cytokines such as IL-1, IL-7, IL-15, IFN-γ, and TNF-α [26]. These cytokines perpetuate chronic inflammation, leading to tissue damage and scarring. Additionally, a reduction in Treg activity exacerbates this immune imbalance, promoting autoimmunity [47,55,56]. Emerging evidence also suggests a role for IL-4/IL-13 signaling in pruritic LS cases, indicating a potential Th2-skewed immune response in certain disease subsets [52]. Nevertheless, a definitive disease marker for LS remains elusive, highlighting the need for further research into the molecular mechanisms that drive disease progression.

Fibrosis, a hallmark feature of LS, is closely linked to TGF-β signaling and ECM remodeling [86,88,91,107]. ECM1, a critical structural and regulatory protein, plays a significant role in LS pathogenesis, as demonstrated by the presence of anti-ECM1 autoantibodies in most patients [77]. Disruptions in ECM1 function lead to altered collagen deposition, compromised elastic fiber integrity, and TGF-β activation, ultimately contributing to sclerosis. Moreover, the interaction between ECM1 and collagen V may form a feedback loop that perpetuates fibrosis [85]. Fibrosis can also impact the penetration of topical therapies, potentially reducing their efficacy. Targeting TGF-β- and ECM1-related pathways offers promising therapeutic potential for managing LS.

Currently available therapies are based on the use of topical corticosteroids and/or calcineurin inhibitors. However, such treatments are not effective in 100% of cases, and alternative strategies for LS are urgently needed. Moreover, dermatologists have raised concerns about using immunosuppressant therapies for LS due to the potential increased cancer risk in patients with LS. In other immune-mediated conditions like psoriasis and atopic dermatitis, the risk of neoplasia is primarily linked to systemic drug-induced suppression of natural anti-cancer immunosurveillance, which is why topical treatments are preferred in high-risk patients [112,113,114,115]. In contrast, LS is associated with cutaneous SCC arising within affected areas, with local immunosuppression potentially exacerbating this risk. Given the long-term malignancy risk in LS-affected skin—particularly SCC—broad-spectrum topical immunosuppressants must be used with caution. However, current evidence suggests that pharmacological disruption of chronic inflammation may halt the initial trigger of LS-induced carcinogenesis, making it largely protective against SCC development. Therefore, targeting specific inflammatory pathways or fibrosis-related mechanisms could offer a safer and more effective therapeutic approach.

Ideally, topical targeted therapies could offer a more selective approach for modulating LS course, reducing the risk of systemic side effects, and improving patient comfort. However, it is important to note that fibrosis may influence the absorption and penetration of topical treatments, potentially affecting their effectiveness.

Despite the significant advancements in understanding LS pathogenesis, several important questions remain unanswered.

The role of infections, particularly *Mycoplasma* and *Borrelia* spp., in disease initiation or exacerbation warrants further investigation. Additionally, conflicting findings regarding the involvement of IL-17 in LS highlight the complexity of its immunopathology [54]. Last but not least, it should be clarified whether anti-fibrotic agents used in systemic sclerosis (e.g., nintedanib and pirfenidone) could be repurposed for LS [116]. Future research should aim to resolve these discrepancies and identify novel therapeutic targets to improve treatment strategies for LS.

## 8. Conclusions

LS presents a significant challenge in both diagnosis and management due to its complex and often subtle clinical presentations. Early and accurate diagnosis, supported by non-invasive techniques, is crucial to prevent irreversible damage and improve patient outcomes. While there is growing evidence for the role of immune dysregulation and fibrosis in LS pathogenesis, further research is needed to unravel the molecular drivers of the disease. Addressing these gaps will be key in developing more effective, targeted therapies that can offer patients better management options and improved quality of life.

## Figures and Tables

**Table 1:** Schematic overview of LS signature cytokines, their production and clinical significance.

Cytokine	Expression	Main Cell Source	Significance	References
IL-1α/β	Increased	Th1 and Th17 cells, macrophages, keratinocytes	Drives inflammation in both innate and adaptive immunity; contributes to fibrosis	Carli et al. [53] Farrell et al. [46] Czajkowski et al. [48] Wang et al. [61]
IL-2	Increased	Th1 cells	Promotes T cell proliferation and immune activation	Ben-Hur et al. [62] Tchórzewski et al. [47] Farrell et al. [46]
IL-7	Increased	Stromal and dendritic cells	Essential for T cell development and survival	Tran et al. [63] De Luca et al. [17]
IL-15	Increased	Dendritic cells, macrophages, epithelial cells	Stimulates NK cells and memory CD8^+^ T cell survival and proliferation	Tran et al. [63] De Luca et al. [17]
IL-17	Increased	Th17 cells, NK cells, γδ T cells	Promotes autoimmunity, activates keratinocytes, and has a controversial role in fibrosis	Dufour et al. [64] Wei et al. [65] Baran et al. [54]
IFN-γ	Increased	Th1 and NK cells	Activates macrophages and other immune cells, sustaining inflammation in LS	Carli et al. [53] Farrell et al. [46] Czajkowski et al. [48]
TNF-α	Increased	Th1 and NK cells, stressed skin cells	Regulates Treg function and immune cell recruitment, driving inflammation	Szabo et al. [66] Farrell et al. [46] Valencia et al. [67] Terlou et al. [51] Gambichler et al. [45]
IL-6	Increased	Th1 cells	Drives inflammation, modulates Treg function, and promotes fibrosis through TGF-β	Romero et al. [68] Farrell et al. [46] Gambichler et al. [45] Johnson et al. [69] Czajkowski et al. [48]
IL-4	Increased *	Th2 cells	Mediates pruritus and fibrosis through Th2 responses and collagen production	Carli et al. [53] Terlou et al. [51] Peterson et al. [52]
IL-10	Decreased	Treg cells	Downstream marker of Treg function, suppressed by the overexpression of miR-155	Corthay [70] Sakaguchi et al. [71] Gambichler et al. [45]
TGF-β	Equal	Th1 cells, Th17 cells, fibroblasts	Key mediator of fibrosis and Treg cell function in skin	Carli et al. [53] Fujimoto et al. [72] Gambichler et al. [45]

* *Controversial among researchers*. Abbreviations: IFN-γ, interferon gamma; IL, interleukin; NK, natural killer cell; TGF-β, transforming growth factor beta; TNF-α, tumor necrosis factor alpha; Th, helper T cell; Treg, T regulatory cell.

## Data Availability

Not applicable.

## Abbreviations

The following abbreviations are used in this manuscript:

ADSC	Adipose-derived stem cell
DEJ	Dermal–epidermal junction
ECM1	Extracellular matrix protein 1
FGF	Fibroblast growth factor
IFN-γ	Interferon gamma
IL	Interleukin
LAP	Latency-associated peptide
LP	Lichen planus
LS	Lichen sclerosus
NK	Natural killer cell
PDGF	Platelet-derived growth factor
PDT	Photodynamic therapy
PLS	Penile LS
PLSCR-1	Phospholipid scramblase 1
PRP	Potential of platelet-rich plasma
RGD	Arginine–glycine–aspartic acid
RT-PCR	Reverse-transcription polymerase chain reaction
SCC	Squamous cell carcinoma
TCIs	Topical calcineurin inhibitors
TCS	Topical corticosteroid
TGF-β	Transforming growth factor-beta
TGFBR	Transforming growth factor-beta receptor
Th	Helper T cells
TNF-α	Tumor necrosis factor alpha
Treg	T regulatory cell
VIN	Vulvar intraepithelial neoplasia
